# The Power of Family Support: The Long-Term Effect of Pre-COVID-19 Family Support on Mid-COVID-19 Work Outcomes

**DOI:** 10.3390/ijerph181910524

**Published:** 2021-10-07

**Authors:** Yuhyung Shin, Won-Moo Hur, Kyungdo Park

**Affiliations:** 1 School of Business, Hanyang University, Seoul 133-791, Korea; yuhyung@hanyang.ac.kr; 2 College of Business Administration, Inha University, Incheon 22212, Korea; 3 Sogang Business School, Sogang University, Seoul 04107, Korea; kyungdo@sogang.ac.kr

**Keywords:** COVID-19, family support, emotional exhaustion, job performance, organizational citizenship behavior

## Abstract

While COVID-19 has triggered a vast amount of research on the effect of the pandemic on employee outcomes, little information is known about how the family-to-work interface affects long-term work outcomes during the pandemic. Drawing on the work–home resources model, this study proposes that family support provided before the onset of COVID-19 has a positive indirect effect on job performance and organizational citizenship behavior (OCB) after the onset, by decreasing emotional exhaustion. To test this proposition, we collected two-wave data from 211 South Korean employees over a 17-month period. As predicted, after controlling for employees’ pre-COVID-19 emotional exhaustion, job performance, and OCB, pre-COVID-19 family support was found to exert a significant indirect effect on mid-COVID-19 job performance (*b* = 0.024, 95% CI = [0.003, 0.071], *ab*_cs_ = 0.027) and OCB (*b* = 0.031, 95% CI = [0.001, 0.084], *ab*_cs_ = 0.033), through mid-COVID-19 emotional exhaustion. This finding suggests that family support has a positive longitudinal effect on work outcomes for employees during the pandemic.

## 1. Introduction

The COVID-19 pandemic has dramatically affected individuals’ work and home lives. Since the outbreak of the pandemic, many countries have implemented teleworking practices to reduce physical contact between individuals [1,2]. However, the proliferation of working from home triggered by the pandemic has resulted in many negative side effects, such as decreased family satisfaction, work–family conflict, isolation from coworkers, work intensification, and decreased opportunities for promotion and career development [3,4]. While a growing body of research has underscored the role of social support in coping with the stress arising from the COVID-19 pandemic [3], relatively scarce attention has been paid to the role of family support (i.e., emotional and instrumental support from family members) in the context of the pandemic. As employees have spent more time with their family members since the COVID-19 outbreak, family has become an important source of social support for those working during the pandemic [4]. Although recent COVID-19 research revealed that family support decreases depression [4], the question of how it affects outcomes in the work domain remains unanswered. To bridge this gap, the present study aimed to examine the effect of family support on employee work outcomes.

Drawing on the work–home resources (WHR) model [5], this study posits that emotional and instrumental support from family members diminishes resource depletion in the work domain, thus contributing to improved work performance. The WHR model further predicts that resources from one domain (either work or home) affect long-term outcomes in the other domain, through changes in personal resources. Based on this model, we propose that family support is positively associated with long-term in-role (task) and extra-role (organizational citizenship) performance by reducing the loss of emotional resources (i.e., emotional exhaustion). By applying this proposition to the context of COVID-19, we further postulate that family support before the onset of COVID-19 helped employees cope with emotional exhaustion during the pandemic and, consequently, helped them perform well in their jobs. This is grounded in the premise that the effect of an external event on the work–family interface should be examined by comparing employees’ responses before and during the event [6]. In particular, given that novel, disruptive, and critical events such as COVID-19 have a massive impact on employee attitudes and behavior [6], the long-term effect of family support can be precisely captured and studied with measures taken before and after the onset of COVID-19. Thus, the purpose of our research was to assess the indirect effect of pre-COVID-19 family support on mid-COVID-19 employee performance, through emotional exhaustion.

Using longitudinal panel data collected before and after the onset of the pandemic, our study aimed to assess the long-term effect of family support on employee in-role and extra-role performance through their level of emotional exhaustion during the pandemic. Our research is expected to make several contributions. First, while COVID-19 research has evaluated family support in the pandemic context, no attempt has yet been made to explore the role of existing family support before the pandemic. By examining the long-term effect of pre-COVID-19 family support on work outcomes after the onset of the pandemic, our research provides a novel understanding of the role of family support. Second, expanding prior studies that focus on workplace support as the antecedent of work outcomes, our research shifts the source of social support from the workplace to the home domain, which contributes to the validation of the WHR model in the pandemic context. Lastly, our research sheds light on the mediating path linking family support and work outcomes. The WHR model theorizes that family support enriches work outcomes not only by providing necessary resources but also by preventing resource depletion. Our research model tests the latter possibility by identifying emotional exhaustion as an intermediary mechanism. 

## 2. Theoretical Background and Hypotheses

### 2.1. The WHR Model

The key tenet of the WHR model is that “contextual home resources improve work outcomes through a gain in personal resources” [5] (p. 549). Since individuals have finite physiological and psychological resources, a surplus of resources in the home (or work) domain spills over into the other area [7]. This model assumes the existence of a work–home interface in which work resources promote home outcomes through personal resources and vice versa [5]. In this model, resources are classified into contextual and social [8]. While the former refers to resources that exist outside the self and can be obtained in the social context of the person (e.g., marriage, employment, home, and social network), the latter encompasses one’s physical, psychological, affective, and intellectual resources (e.g., positive emotions, self-esteem, resilience, and energy [8]). 

The WHR model postulates contextual resources to be the starting point in initiating work–home or home–work enrichment processes [5]. Put differently, contextual resources in the work domain (e.g., a pay raise) improve employees’ personal resources, facilitating outcomes in the home domain (e.g., relationship with spouse). Likewise, contextual resources in the home domain (e.g., support from family members) enrich personal resources. These resources, when used in the work domain, lead to increased work engagement and performance [5]. Drawing on the home-to-work process proposed by the WHR model, we contend that family support serves as a key contextual resource that facilitates work.

According to the WHR model, the development of personal resources in one domain prevents the depletion of resources in the other domain [5]. Applying this proposition to the home-to-work process, family support should provide employees with personal resources to buffer them against emotional exhaustion at work. Thus, a reduced level of emotional exhaustion is associated with increased work performance. 

The WHR model further proposes that chronic contextual resources in one domain (e.g., family support) encourage individuals to achieve long-term goals in the other by providing sustained personal resources [5]. Based on this theory, family support is posited to exert a long-term positive effect on employees’ work performance by offering them sustained resources and energies, preventing them from becoming exhausted at work and enabling them to accomplish their performance goals. As a result, employees who receive support from family members will display increased in-role and extra-role performance in the long run.

### 2.2. Hypothesis Development

The social support literature categorizes social support into emotional and instrumental [9]. The former refers to showing sympathy, concern, respect, or encouragement toward others, whereas the latter pertains to providing tangible or physical aid [9]. When applied to the family domain, instrumental support refers to family members’ behavior, involving sharing household tasks (e.g., running errands, tidying the house, and taking the children to school) [10].

Research on family support in non-pandemic settings indicates that support from family decreases family interference in work [11,12,13] and reduces work–family conflict [14] by clarifying work and family roles and enhancing the work–family balance. Moreover, family support is found to provide employees with social and emotional resources, which protects them from feeling emotionally exhausted at work [15]. Emotional exhaustion, a core dimension of burnout, refers to feeling emotionally fatigued and drained [16]. We assert that emotional exhaustion is a key mediator linking family support and performance outcomes. Furthermore, we consider job performance and organizational citizenship behavior (OCB) as important work performance outcomes. Job performance is defined as the extent to which an employee successfully fulfills his/her work duties and responsibilities [17], while OCB refers to the discretionary behavior one undertakes beyond one’s formal job descriptions, which is helpful to others in the workplace [18]. As job performance and OCB represent two distinct aspects of overall work performance, we identify them as key work outcomes [19].

Drawing on the WHR model, we propose that family support which existed before the outbreak of COVID-19 contributes positively to the job performance and OCB of employees during the pandemic, by reducing their emotional exhaustion. Both job performance and OCB require substantial physical and psychological resources. To perform their job, employees must have attentional resource capacity and self-regulatory resources [20]. As OCB involves expending extra effort beyond one’s in-role duties, it necessitates additional resources [19]. We propose that emotional and instrumental resources from family members spill over to the work domain by providing employees with the psychological and physical resources necessary for job performance and OCB. As proposed by the WHR model, emotional and instrumental forms of support from family members serve as contextual resources that promote positive emotions, self-esteem, vigor, and resilience [21,22]. These psychological resources act as catalysts that connect the home and work domains. In sum, positive psychological resources gained through family support buffer employees against emotional exhaustion at work. Furthermore, employees who receive emotional support from their families are likely to effectively regulate their emotions, and thus, experience less emotional fatigue, even in the face of the stressors resulting from COVID-19 [23]. Additionally, instrumental support from family members alleviates physical and mental demands at home, thereby preventing employees from feeling exhausted at work.

According to the WHR model, contextual resources in one domain enable employees to attain long-term goals in the other by accumulating personal resources over time [5]. Compared with the work domain, in which employees’ interaction partners (e.g., coworkers and customers) often change, families do not undergo frequent membership changes. Such chronic contextual resources (e.g., family support) provide individuals with personal resources that help them to cope with an unexpected environmental crisis (e.g., the COVID-19 pandemic). Therefore, family support functions as a chronic contextual resource in the pandemic context, creating a gain spiral in which resources are accumulated over a long-term span [24]. When employees received emotional and instrumental support from family before the onset of COVID-19, the personal resources shaped before the pandemic cushioned employees against the negative consequences of the pandemic and work stressors (e.g., fear of COVID-19 infection, potential job loss, and isolation from coworkers), and would help prevent them from feeling emotionally drained in the workplace. Diminished emotional exhaustion provides employees with more psychological resources to guard against future emotional exhaustion [25]. As a result, family support is posited to decrease emotional exhaustion over time.

The negative relationships between emotional exhaustion and job performance, and emotional exhaustion and OCB, have been well established [25,26,27]. Since job performance and OCB require considerable physical and psychological resources, a state of resource depletion, such as emotional exhaustion, is expected to undermine the two performance outcomes. Emotional exhaustion decreases job performance by depriving employees of mental resources and energy needed to perform various tasks effectively. It dampens employees’ motivation to achieve their performance goals and dissuades them from staying focused at work; this, consequently, results in decreased job performance [27]. Similarly, emotional exhaustion is associated with diminished OCB by dampening employees’ social motivation [27] and weakening their commitment toward their organization [25]. In particular, as extra-role behavior demands additional resources, beyond those necessary for in-role performance [19], emotionally exhausted employees are unlikely to engage in OCB. Conversely, employees who are not emotionally exhausted have greater resources that they can then invest in work activities, and maintain high levels of job performance and OCB.

Taken together, we postulate a mediating relationship, in which pre-COVID-19 family support is negatively associated with mid-COVID-19 job performance and OCB through emotional exhaustion. As predicted by the WHR model, pre-COVID-19 family support serves as a chronic contextual resource for employees working during the pandemic. As they are equipped with positive personal resources (e.g., emotional stability, confidence, optimism, and resilience), they are likely to experience less emotional exhaustion amid COVID-19. Thus, they continue exerting effort and energy in their in-role and extra-role activities during such a crisis and exhibit high levels of job performance and OCB. This line of reasoning leads to the following mediation hypotheses:

**Hypothesis** **1.** *Pre-COVID-19 family support has an indirect effect on mid-COVID-19 job performance through emotional exhaustion*.

**Hypothesis** **2.** *Pre-COVID-19 family support has an indirect effect on mid-COVID-19 OCB through emotional exhaustion.*

## 3. Method

### 3.1. Data Collection Procedure and Sample Characteristics

To enhance the generalizability of this study’s findings, we gathered data from 211 individuals employed in various organizations (e.g., airlines, banks, and retail stores) located in South Korea. The first cases of COVID-19 were found in the country on 20 January 2020. Despite social distancing policies and vaccinations, the number of confirmed COVID-19 cases continues to increase in South Korea. As of August 2021, South Korea has more than 200,000 confirmed cases, with its first, second, and third wave of the pandemic occurring in March, August, and December 2020, respectively. To examine the long-term effect of family support on employee work outcomes amid COVID-19, we measured respondents’ family support, emotional exhaustion, job performance, OCB, and control variables (i.e., demographic characteristics and positive and negative affectivity) at two points in time by administering a pre-COVID-19 survey in July 2019 (Time 1: T1) and a mid-COVID-19 survey in December 2020 (Time 2: T2).

Survey invitations were distributed to full-time employees registered on an online survey platform operated by a South Korean research company. After they agreed to the informed consent form, which guaranteed anonymity and confidentiality, we emailed them an online survey link. Of the 651 individuals who filled out the initial T1 survey, 211 participated in the T2 survey (retention rate = 32.4%). We checked the optimal sample size with the G*Power sample size calculator [28]. According to this calculation, the optimal sample size for a power of 0.90 with a medium effect size (i.e., 0.15) and a significance level of 0.05 [29] should range between 153 and 160. Exceeding this criterion, our sample size (N = 211) can be considered to be an optimal sample size. A total of 59% of the participants were female; the average age and job tenure of the respondents was 35.86 (*SD* = 8.13) and 5.29 (*SD* = 4.74) years, respectively. The majority of the respondents had a four-year university education (47.4%), followed by a two-year college education (18.5%), a graduate-level education (2.4%), and a high school education (31.8%).

To check the equivalence of the final sample and those who dropped out at T2, we compared the differences between the final sample (*N* = 211) and the dropouts (*N* = 440) using a series of *t*-tests [30]. As there were no significant differences in family support, emotional exhaustion, job performance, OCB, and control variables between these two groups, we ascertained that our data were not affected by non-response bias (see Table 1).

### 3.2. Measures

We followed Brislin’s [31] back-translation procedure to construct the survey items. All variables were measured on a five-point Likert-type scale ranging from 1 to 5 (1 = “strongly disagree”, 5 = “strongly agree”) (see Table 2). Family support was evaluated using the six-item family support scale of King et al. [10] (e.g., “When I succeed at work, members of my family show that they are proud of me” and “Someone in my family helps me out by running errands when necessary”). Emotional exhaustion was assessed using four items from the Maslach Burnout Inventory [16] (e.g., “I feel emotionally drained from my work”). We asked the respondents to rate their own job performance and OCB by using four items from Williams and Anderson’s in-role performance scale [17] (e.g., “I meet the formal performance requirements of my job”) and seven items from Van Dyne and LePine’s OCB scale [32] (e.g., “I help coworkers learn about the work”).

We controlled for the respondents’ age, gender, job tenure, and positive and negative affectivity in all subsequent analyses due to their potential effects on emotional exhaustion, job performance, and OCB (e.g., [33,34,35]). We used the Positive Affect and Negative Affect Schedule Short Form [36] to assess positive and negative affectivity.

### 3.3. Analytic Strategy

To estimate the proposed indirect effects, we employed path modeling and bootstrapping (*N* = 5000) with the M-plus-based PROCESS macro [37,38]. To precisely capture the longitudinal effects of T1 family support on emotional exhaustion, job performance, and OCB at T2, we included a path from T1 emotional exhaustion to T2 emotional exhaustion; a path from T1 job performance to T2 job performance; and a path from T1 OCB to T2 OCB [39,40]. Furthermore, we estimated the effect size of the proposed model using the standardized indirect effect (*ab*_cs_; [41]). The standardized indirect effects of 0.01, 0.09, and 0.25 indicate small, medium, and large effect sizes, respectively [42].

## 4. Results

### 4.1. Reliability, Validity, and Descriptive Statistics

Table 3 reports the descriptive statistics and correlations. Definitions of the mean, standard deviation, and correlation are provided in the footnote of Table 3. For example, the mean age was calculated using this following formula: *mean*_age_ = (age _id1_ + age _id1_ + age _id1_ … + age _id221_)/221. All reliability indices met the criterion for high reliability [43]. The proposed nine-factor model (i.e., family support, positive and negative affectivity (T1), emotional exhaustion, job performance, and OCB (T1 and T2)) exhibited an acceptable fit in an absolute sense: (χ ^2^_(783)_ = 1405.90, *p* < 0.05, comparative fit index (CFI) = 0.91, Tucker Lewis index (TLI) = 0.90, root mean square error of approximation (RMSEA) = 0.06, standardized root mean square residual (SRMR) = 0.05). Furthermore, family support, emotional exhaustion, job performance, and OCB possessed a sufficient level of composite reliability, ranging from 0.84 to 0.94. All the average variance extracted values were larger than the squared correlation between the target construct and any of the other constructs [44].

### 4.2. Hypothesis Testing

**Figure 1:**
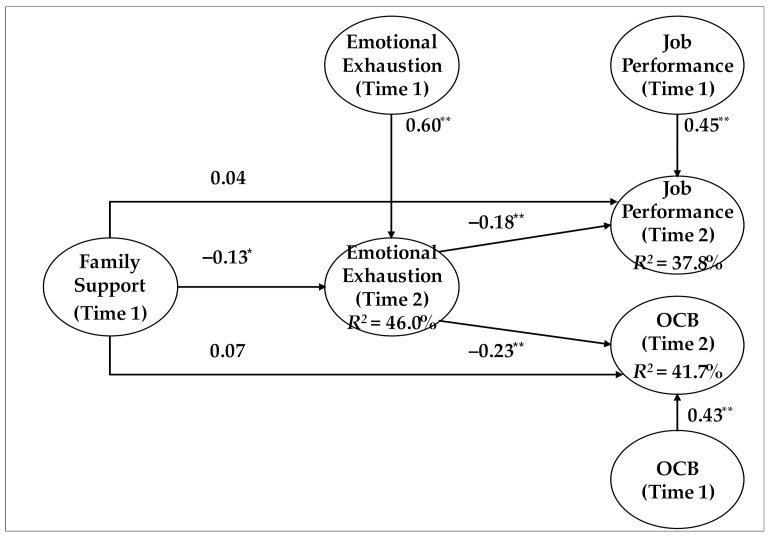
Summary of results. Notes: Unstandardized coefficients are reported. OCB = organizational citizenship behavior. For parsimony, the results for the control variables are omitted. * *p* < 0.05, ** *p <* 0.01. *R*^2^: a statistical measure that represents the proportion of the variance for a dependent variable (range: minimum: 0; maximum: 1).

Hypothesis 1 postulated that emotional exhaustion would mediate the relationship between family support and job performance. As shown in Table 4 and Figure 1, even after controlling for emotional exhaustion and job performance at T1, T1 family support exerted a significant indirect effect on T2 job performance through T2 emotional exhaustion (*b* = 0.024, 95% CI = [0.003, 0.071], *ab*_cs_ = 0.027). Thus, Hypothesis 1 was supported.

Hypothesis 2 proposed a mediating effect of emotional exhaustion on the family support–OCB relationship. In support of this hypothesis, T1 family support had a significant indirect effect on T2 OCB through T2 emotional exhaustion, even after controlling for emotional exhaustion and OCB at T1 (*b* = 0.031, 95% CI = [0.001, 0.084], *ab*_cs_ = 0.033).

## 5. Discussion

### 5.1. Theoretical Implications

The findings of the present study demonstrate that family support before the onset of COVID-19 exerted a positive indirect effect on job performance and OCB after its onset, by decreasing emotional exhaustion; that is, even after controlling for emotional exhaustion, job performance, and OCB before the onset of COVID-19, pre-COVID-19 family support significantly predicted emotional exhaustion, job performance, and OCB after its onset. As such, family support was sufficiently powerful to promote job performance and OCB during the pandemic. These findings corroborate the key proposition of the WHR model—that home resources spill over to the work domain, thereby contributing positively to work outcomes. Our findings further endorse the WHR proposition, which asserts that stable contextual resources, such as family support, have a long-term effect on work outcomes through the accumulation of resources. Taken together, our findings suggest that the WHR model is a pertinent framework to understand employees’ work–family interface during the pandemic.

Our research expands the extant body of research on the work–family interface amid COVID-19 by assessing the longitudinal effect of pre-COVID-19 family support. The majority of COVID-19 research has focused on the negative effect of the pandemic on family outcomes, based on data collected after its onset. To the best of our knowledge, virtually no studies have explored whether family support before the onset of COVID-19 protects employees from emotional exhaustion after its onset. In response to the call from Vaziri et al. [6] for pre- and mid-COVID-19 measurements, by assessing the study variables both before and during COVID-19, we found that pre-COVID-19 family support accounted for changes in emotional exhaustion, job performance, and OCB over a 17-month period.

We further advanced COVID-19 research by examining the source of social support from the home rather than the work domain. Complementing prior research that highlights the buffering role of supervisor or coworker support amid COVID-19 [45], our research unraveled the role of family support in such crises. As such, our findings provide novel insights into the role of family support in the context of the pandemic by demonstrating family support as a coping mechanism that alleviates emotional exhaustion and enhances job performance and OCB.

### 5.2. Practical Implications

Our findings have managerial implications for organizations that strive to maintain productivity during the COVID-19 crisis. Based on our finding that family members with pre-existing sources of strength and support from family are less emotionally exhausted and perform better during the pandemic, it is critical to build supportive relationships among family members [46]. Heightening family members’ awareness that emotional and instrumental support benefits other members’ performance in the workplace would be a first step toward such an endeavor. Moreover, family counseling can be used for facilitating communication and conflict resolution between family members. Given that work and home issues are inseparable among employees working during COVID-19, organizations need to cultivate a family-supportive environment. Organizations can consider implementing supportive policies and flexible work arrangements for employees experiencing family-to-work interference [7,47]. Finally, as family-supportive policies and culture cannot be realized without supervisors’ participation, supervisors should take a proactive stance in creating a family-supportive work environment. They need to understand and sympathize with employees with family issues and help them handle such problems [7]. Family-supportive initiatives implemented by organizations and supervisors will help employees maintain a healthy work–family balance, which in turn will contribute to their long-term performance, even during the COVID-19 crisis.

### 5.3. Limitations and Directions for Future Research

Despite the use of longitudinal data, the present findings should be interpreted in light of the following limitations. First, self-reported performance measures are vulnerable to social desirability and common method variance (CMV). CMV refers to “variance that is attributable to the measurement method rather than to the constructs the measures represent” [48] (p. 879). When the independent variable, mediator, and dependent variable are measured by the same source, respondents’ response tendency may inflate the relationships between these variables [48]. Although time-separated measurements employed in our research reduce the risk of CMV [48], reliance on single-source data might have inflated the relationships among family support, emotional exhaustion, job performance, and OCB. We therefore recommend that future studies use multi-source data (e.g., others’ ratings of job performance and OCB). 

Second, while we argued that family support would have a positive effect on work outcomes by expanding personal resources, we neither measured nor proposed the role of personal resources. Although emotional exhaustion represents a lack of personal resources, the personal resource that plays a mediating role between family support and work outcomes remains unknown. Moreover, it is plausible that the support provided by family members before the onset of the pandemic predicts family support after its onset, which in turn affects work outcomes during the pandemic. As such, T2 family support can mediate the relationship between T1 family support and T2 work outcomes. Unfortunately, the lack of T2 family support data did not allow us to test its mediating effect. Toward a more elaborate understanding of the home-to-work interface, we encourage future researchers to explore the intervening mechanisms of personal resources and family support that are shaped amid the pandemic. 

Third, whilst Aguinis et al. [49] stated that even an effect size of 1% can be practically and scientifically important, and our findings show that the mediation effects were robust (i.e., 2.7% and 3.3%), it should be noted that the relatively small sample size increased the risk of falsely detecting a significant relationship. To reduce this risk, future research should use a larger sample. In addition, although we drew our sample from diverse organizations to enhance the generalizability of the study findings, our sample consisted of only South Korean employees. Thus, the present findings need to be cross-validated among employees in different countries. 

Fourth, our research did not consider family-related variables (e.g., dual-earner couples, family size, number and age of children, and family income [50,51]) in assessing the role of family support. Given the potential interplay between these variables and family support [52,53], future research needs to take these factors into account to precisely capture the effect of family support on work outcomes. In addition, COVID-19 research has demonstrated that the conditions of habitability in the home (e.g., telework space) affect employees’ resilience and satisfaction [54,55]. Based on these findings, the potential effect of the conditions of habitability in homes and telework-related variables on family support should be delved into in future research. 

Lastly, while we focused on family support as a crucial form of social support, other forms of support (e.g., organizational and supervisory support) can also be an important source of social resources among employees during the COVID-19 pandemic. Considering the fact that employees interact with their supervisors and coworkers during working hours, support from them can also help to buffer employees against emotional exhaustion. For this reason, we recommend that future social support research simultaneously examine the relative roles of different forms of social support in the home and work domains. 

## 6. Conclusions

Despite a vast amount of research on COVID-19, relatively little attention has been paid to the long-term effect of family support in coping with the pandemic. The present study aimed to test the indirect effect of pre-COVID-19 family support on mid-COVID-19 job performance and OCB through emotional exhaustion. As predicted, pre-COVID-19 family support exerted a significant indirect effect on mid-COVID-19 job performance (*b* = 0.024, 95% CI = [0.003, 0.071], *ab*_cs_ = 0.027) and OCB (*b* = 0.031, 95% CI = [0.001, 0.084], *ab*_cs_ = 0.033) through emotional exhaustion. These findings highlight the importance of family support in the time of the pandemic by demonstrating that pre-COVID-19 family support was sufficiently powerful to predict employees’ emotional exhaustion, work performance, and OCB. Our research provides novel insights into the role of family support by demonstrating family support to be a coping mechanism that mitigates emotional exhaustion and enhances job performance and OCB amid the pandemic. Furthermore, by validating the WHR model in the context of the COVID-19 pandemic, our research makes a theoretical contribution to the literature on the work–home interface. Follow-up research into the interplay between family support and other family- and habitability-related variables could elaborate on the insights gained from the present findings.

## Figures and Tables

**Table 1:** The results of the *t*-test before and after the onset of COVID-19.

Variables	Mean for Final Sample (*N* = 211)	Mean for Drop-Out Sample (*N* = 440)	*t*-Value
Family Support	3.78	3.75	0.48 (*p* = n.s.)
Emotional Exhaustion	2.23	2.20	0.40 (*p* = n.s.)
Job Performance	3.88	3.94	0.99 (*p* = n.s.)
OCB	3.56	3.59	0.58 (*p* = n.s.)
Positive Affectivity	2.45	2.34	1.42 (*p* = n.s.)
Negative Affectivity	2.85	3.97	1.46 (*p* = n.s.)

**Table 2:** Measurement items and factor loadings.

Construct	Measurement Items	T1	T2
Family Support	Members of my family want me to enjoy my job.	0.65	-
	Members of my family are happy for me when I am successful at work.	0.90	-
	When I succeed at work, members of my family show that they are proud of me.	0.93	-
	Someone in my family helps me out by running errands when necessary.	0.68	-
	Members of my family are willing to straighten up the house when it needs it.	0.62	-
	Members of my family cooperate with me to get things done around the house.	0.66	-
Emotional Exhaustion	I feel emotionally drained from my work.	0.51	0.62
	I feel fatigued when I get up in the morning and have to face another day on the job.	0.82	0.88
	Working with people all day is really a strain for me.	0.91	0.92
	I feel burned out from my work.	0.86	0.88
Job Performance	I adequately complete assigned duties.	0.84	0.87
	I perform tasks that are expected of me.	0.89	0.89
	I fulfill the responsibilities specified in my job description.	0.82	0.83
	I meet the formal performance requirements of my job.	0.81	0.82
OCB	I assist co-workers in this group with their work for the benefit of the group.	0.80	0.80
	I help co-workers in this group learn about the work.	0.83	0.81
	I help co-workers in this group with their work responsibilities.	0.79	0.80
	I get involved to benefit group work.	0.83	0.80
	I help others in this group learn about the work.	0.85	0.87
	I help orient new employees in this group.	0.64	0.67
	I attend functions that help this group.	0.86	0.87
Positive Affectivity	Alert	0.70	-
	Inspired	0.78	-
	Active	0.89	-
Negative Affectivity	Nervous	0.75	-
	Upset	0.95	-
	Ashamed	0.82	-

Notes: Items measured on a scale ranging from 1, “strongly disagree,” to 5, “strongly agree.”.

**Table 3:** Means, standard deviations, and correlations.

Variables	M	SD	α	CR	1	2	3	4	5	6	7	8	9	10	11	12
1. Gender	0.41	0.49	-	-	-											
2. Age	35.86	8.13	-	-	0.12	-										
3. Job tenure	5.29	4.74	-	-	0.05	0.50 **	-									
4. Positive affectivity (T1)	2.45	0.87	0.83	0.84	0.14 *	0.08	−0.13	**0.63**								
5. Negative affectivity (T1)	2.85	0.98	0.88	0.88	−0.09	−0.27 **	−0.12	−0.25 **	**0.71**							
6. Family support (T1)	3.78	0.74	0.89	0.88	−0.04	−0.01	0.05	0.18 **	−0.23 **	**0.56**						
7. Emotional exhaustion (T1)	2.23	0.83	0.86	0.87	0.08	−0.00	0.05	−0.20 **	0.34 **	−0.37 **	**0.63**					
8. Emotional exhaustion (T2)	2.31	0.88	0.90	0.90	0.05	−0.12	−0.07	−0.16 *	0.36 **	−0.35 **	0.65 **	**0.70**				
9. Job performance (T1)	3.88	0.65	0.91	0.91	−0.06	−0.05	−0.12	0.18 **	−0.11	0.33 **	−0.42 **	−0.43 **	**0.71**			
10. Job performance (T2)	3.87	0.68	0.91	0.91	−0.10	−0.11	−0.17 **	0.18 **	−0.09	0.27 **	−0.41 **	−0.42 **	0.57 **	**0.73**		
11. OCB (T1)	3.56	0.68	0.92	0.94	−0.10	−0.03	−0.03	0.14 *	−0.14 *	0.42 **	−0.34 **	−0.35 **	0.47 **	0.42 **	**0.69**	
12. OCB (T2)	3.56	0.70	0.93	0.93	−0.18 **	0.02	0.02	0.18 **	−0.03	0.33 **	−0.29 **	−0.43 **	0.34 **	0.54 **	0.59 **	**0.65**

Notes:  *N* = 211. Bold numbers along the diagonal are average variance extracted values. OCB = organizational citizenship behavior; T1 = time 1 (July 2019); T2 = time 2 (December 2020); CR = composite reliability. Gender: 0 = female; 1 = male. * p < 0.05, ** p < 0.01. Formulae: $Mean=\bar{x}=\frac{\sum_{i}^{n}x_{i}}{n}$. $Standard\;deviation=SD=\frac{\sum_{i}^{n}\left(x-\bar{x}\right)}{n}$. $Correlation=\frac{\sum_{i}^{n}\left(Z_{X}Z_{Y}\right)}{n}$.

**Table 4:** Path coefficients and mediation indices.

Variable	Emotional Exhaustion (T2)	Job Performance (T2)	OCB (T2)
	*b*	*b*	*b*
Gender	0.03	−0.08	−0.18 *
Age	−0.01	−0.01	0.00
Job tenure	−0.01	−0.01	0.01
Positive affectivity (T1)	−0.01	0.05	0.10 *
Negative affectivity (T1)	0.10 *	0.03	0.13 **
Family support (T1)	−0.13	0.04	0.07
Emotional exhaustion (T1)	0.60 **		
Emotional exhaustion (T2)		−0.18 **	−0.23 **
Job performance (T1)		0.45 **	
OCB (T1)			0.43 **
*R* ^2^	46.0%	37.8%	41.7%
Mediation Indices			
Family Support → Emotional exhaustion → Job performance: *b* = 0.024, 95% CI = [0.003, 0.071]			
Family Support → Emotional exhaustion → OCB: *b* = 0.031, 95% CI = [0.001, 0.084]			

Notes: *b =* unstandardized coefficient. OCB = organizational citizenship behavior; T1 = time 1; T2 = time 2. Gender: 0 = female; 1 = male. * *p* < 0.05, ** *p* < 0.01.

## Data Availability

The data presented in this study are available upon request from the corresponding author.
